# Efficacy of non-pharmacological treatments for prolonged disorders of consciousness: a network meta-analysis of randomized controlled trials

**DOI:** 10.3389/fneur.2026.1754777

**Published:** 2026-05-13

**Authors:** Chunyue Xu, Han Yang, Wenyue Cai, Qun Liu, Mingjia Zhang, Hongge Zuo, Jianbang Su, Jingqi Shu, Zhenhua Xu

**Affiliations:** 1 The Second Clinical Medical College, Guangzhou University of Chinese Medicine, Guangzhou, Guangdong Province, China; 2 Acupuncture, Moxibustion, and Rehabilitation Clinical Medical College, Guangzhou University of Chinese Medicine, Guangzhou, China

**Keywords:** network meta-analysis, neurorehabilitation, non-pharmacological treatments, prolonged disorders of consciousness, randomized controlled trials

## Abstract

**Objective:**

To explore the efficacy of non-pharmacological treatments such as repetitive transcranial magnetic stimulation (rTMS), transcranial direct current stimulation (tDCS), median nerve stimulation (MNS), hyperbaric oxygen (HBO), and acupuncture in improving the level of consciousness in patients with prolonged disorders of consciousness (pDOC).

**Methods:**

PubMed, Embase, Cochrane Library, Web of Science, CNKI, Wanfang, China Biology Medicine (CBM), and VIP Database were systematically searched from inception to December 2024. Data synthesis and visualization were conducted using the “coda” and “gemtc” packages in R software and STATA 17.0. The Jadad scale was used for initial screening to exclude low-quality studies, and the Cochrane Risk of Bias 2.0 tool was applied to assess the methodological quality of included randomized controlled trials (RCTs).

**Results:**

A total of 32 randomized controlled trials (RCTs) enrolling 1,770 participants were included in this network meta-analysis. The pooled results demonstrated that rTMS, tDCS, MNS, HBO, and acupuncture were all associated with improved scores on the Coma Recovery Scale-Revised (CRS-R). The mean difference (MD) ranged from 17.32 (95% CrI: 6.57 to 104.25) in the rTMS group to 3.56 (95% CrI: 0.61 to 40.45) in the acupuncture group. According to the Surface Under the Cumulative Ranking Curve (SUCRA), rTMS was associated with the highest probability of being the most effective intervention. In subgroup analyses, among patients with minimally conscious state (MCS), the MD ranged from 15.99 (95% CrI: 1.57 to 66.77) in the tDCS group to 9.72 (95% CrI: 2.07 to 61.04) in the MNS group, and among patients with unresponsive wakefulness syndrome (UWS), the MD ranged from 18.52 (95% CrI: 2.15 to 108.73) in the rTMS group to 4.12 (95% CrI: 0.25 to 69.30) in the acupuncture group.

**Conclusion:**

rTMS, tDCS, MNS, HBO, and acupuncture may improve CRS-R scores and promote consciousness recovery in patients with pDOC. Among these interventions, tDCS may be associated with more favorable effects in patients with MCS, whereas rTMS appears to be more beneficial for those with UWS.

## 1 Introduction

As neurocritical care advances, more patients survive severe brain injury, contributing to the rising global prevalence of prolonged disorders of consciousness (pDOC) (1). pDOC is defined as a state of sustained consciousness impairment lasting ≥28 days after severe acquired brain injury and is mainly categorized into unresponsive wakefulness syndrome (UWS; formerly vegetative state, VS) and minimally conscious state (MCS). Patients with UWS and MCS present preserved wakefulness but impaired awareness, consistent with the classic arousal-awareness model of consciousness classification (2). The major etiologies of pDOC include traumatic brain injury (TBI), stroke, and hypoxic-ischemic encephalopathy (3). These conditions lead to widespread network dysfunction, which specifically involves the ascending reticular activating system (ARAS) responsible for arousal regulation, thalamocortical circuits, corticocortical connections, corticosubcortical networks, and core hubs of the default mode network associated with conscious awareness (4, 5). In addition, patients often develop severe complications such as infections, pressure ulcers, epilepsy, limb spasticity, and malnutrition, which may further aggravate neurological injury and hinder long-term rehabilitation (6). However, accurate evaluation and differential diagnosis of consciousness levels remain challenging, clinical outcomes vary substantially across individuals, evidence for effective targeted interventions remains limited, and long-term care imposes substantial physical, psychological, and economic strain on families and society (7). Accordingly, pDOC has become an increasingly urgent public health issue that urgently requires evidence-based management strategies (8).

Currently, evidence supporting pharmacological interventions for pDOC remains limited, inconsistent, and lacks high-quality verification (9). Dopaminergic agents are the most extensively studied category, yet their definitive efficacy in pDOC populations has not been established (10). Amantadine, a representative dopaminergic drug, is one of the few guideline-recommended pharmacotherapies for TBI-related pDOC, yet its clinical application is limited by notable constraints. Furthermore, long-term administration may lead to reduced efficacy over time and carries well-documented safety risks in pDOC patients, including seizures, paralytic ileus, and tachycardia (11). Efficacy results are highly inconsistent across studies due to methodological flaws, small sample sizes, and insufficient outcome assessments. High-quality evidence for non-TBI-related pDOC is scarce, and its efficacy remains unclear (12). Zolpidem, another widely investigated agent for pDOC, also presents considerable limitations, with highly variable efficacy, modest overall effects, and most supporting evidence remaining preliminary (13, 14).

Given the substantial limitations of pharmacotherapy, non-pharmacological interventions have become important components of pDOC management and have been gaining growing clinical and research attention (15, 16). rTMS, tDCS, MNS, HBO, and acupuncture are widely used in clinical practice and supported by relevant clinical studies and guidelines (17–22). Patients with pDOC exhibit disrupted connectivity within consciousness-related networks, and these interventions may modulate such networks through distinct direct or indirect pathways (22, 23). The Coma Recovery Scale-Revised (CRS-R), as the gold standard for behavioral assessment in pDOC, is commonly used as the primary outcome measure to evaluate treatment-related changes in the level of consciousness (24). rTMS, commonly applied to the dorsolateral prefrontal cortex (DLPFC), is hypothesized to facilitate recovery of consciousness by modulating cortical excitability, influencing thalamocortical functional connectivity, and enhancing neuroplasticity (25). Previous studies suggest that rTMS may improve CRS-R scores in patients with pDOC (26). tDCS modulates the resting membrane potential of cortical neurons, thereby influencing cortical excitability in the stimulated regions and associated brain areas. It may also affect regional cerebral blood flow and functional connectivity, including within the default mode network and frontoparietal networks (27). Anodal tDCS over the DLPFC has been shown to improve CRS-R scores in patients with MCS, with a favorable tolerability profile (28). MNS activates the brainstem reticular formation, thalamic intralaminar nuclei, and widespread cortical arousal regions via peripheral afferent pathways, thereby facilitating signal transmission within the ARAS. This technique is feasible for bedside application, and meta-analyses suggest that MNS may contribute to recovery of consciousness (20). HBO may exert effects on damaged thalamocortical circuits by increasing local oxygen supply, reducing cerebral edema, inhibiting inflammatory injury, and promoting neuroplastic repair (29). While some studies suggest that HBO may improve levels of consciousness, its overall effect size and clinical utility remain to be validated by high-quality studies. Acupuncture may influence neural information transmission through stimulation of specific acupoints, which is hypothesized to activate the ARAS and limbic-thalamocortical pathways. Preliminary findings indicate that acupuncture combined with conventional rehabilitation may have a beneficial effect on CRS-R scores in patients with pDOC, although current evidence remains limited (22, 30). Despite the potential benefits of these interventions in pDOC rehabilitation, the existing evidence is largely derived from pairwise meta-analyses of single interventions and is constrained by small sample sizes, methodological heterogeneity, and a lack of head-to-head comparisons. Consequently, the comparative efficacy and clinical hierarchy of these interventions remain to be fully elucidated.

Therefore, the present study systematically collected and analyzed controlled trials of rTMS, tDCS, MNS, HBO, and acupuncture in patients with pDOC to conduct a network meta-analysis. Using changes in total CRS-R scores as the primary outcome measure, we compared the efficacy of these interventions for improving the level of consciousness and investigated a preliminary hierarchy of clinical efficacy. The findings may provide evidence-based support for individualized rehabilitation decision-making, optimize therapeutic selection, improve clinical resource allocation, and help prioritize future research directions for pDOC rehabilitation.

## 2 Methods

### 2.1 Study registration

This systematic review followed the PRISMA guidelines (31) and adhered to the registered protocol available on the PROSPERO platform (ID: CRD42025635043).

### 2.2 Search strategy

A comprehensive search was conducted in four English databases, including PubMed, Cochrane Library, Web of Science, and EMBASE, as well as four Chinese databases, including China Biology Medicine, CNKI, VIP Database, and Wanfang Data, as of December 2024. The search terms for intervention measures included “repetitive transcranial magnetic stimulation”, “transcranial direct current stimulation”, “median nerve stimulation”, “acupuncture” and “hyperbaric oxygen therapy”. For the pathology, terms such as “disorders of consciousness”, “unresponsive wakefulness syndrome”, “vegetative state”, “minimally conscious state” were used. Two researchers (XCY and CWY) independently conducted the searches, screened titles and abstracts, excluded studies that did not meet the inclusion criteria, and invited a third researcher (YH) to discuss and resolve differences. A customized search strategy was used for each database.

### 2.3 Inclusion and exclusion criteria

Inclusion criteria were established using the PICOS framework: (P) Patients aged ≥18 years with a diagnosis of pDOC; (I) Interventions including rTMS, tDCS, MNS, acupuncture, and HBO; (C) No specific requirements for the control group; (O) The mean change in CRS-R score from baseline to the end of the last treatment session was defined as the primary outcome measure; (S) Randomized controlled trials with parallel or crossover designs. Exclusion criteria were as follows: (1) reviews, conference papers, or studies published only in abstract form; (2) studies with missing or incomplete data that prevented data extraction; (3) duplicate publications.

### 2.4 Literature selection and data extraction

After completing the literature search, all records were imported into EndNote 20 for deduplication. Preliminary screening was performed by reviewing titles and abstracts to exclude irrelevant literature. Full-text articles were then obtained and further evaluated to exclude studies without available full text or those that did not meet the predefined inclusion criteria. Data extraction was independently conducted by two reviewers (LQ and YH). The following data were extracted: first author, publication year, country, study design, patient age, sample size, gender ratio, disease duration, intervention measures, treatment duration, and outcome measures.

### 2.5 Quality assessment

The methodological quality of the included studies was independently evaluated by two reviewers (ZMJ and ZHG) using the modified Jadad scale (32, 33). Studies with a score ≥4 were considered high quality and included in the analysis. In addition, the Cochrane Risk of Bias tool was used to assess the following domains: random sequence generation, allocation concealment, blinding of participants and personnel, blinding of outcome assessment, incomplete outcome data, selective reporting, and other sources of bias. Each domain was rated as low risk, high risk, or unclear risk of bias.

### 2.6 Statistical analysis

All statistical analyses were performed using R software within the RStudio integrated development environment. The “coda” and “gemtc” packages were employed for model estimation. We conducted a Bayesian network meta-analysis to quantify between-study heterogeneity and derive probability rankings for each intervention. Treatment hierarchy was quantified using the surface under the cumulative ranking curve (SUCRA), where values closer to 1 indicate a higher probability of being the most effective intervention. Effect sizes were calculated as mean differences (MD) for continuous outcomes. To evaluate the reliability of our estimates, we used 95% credible intervals (CrI). Two models were fitted: a fixed-effects model and a random-effects model. Specifically, when the random-effects model exhibited a lower Deviance Information Criterion (DIC) and fewer outliers, it was selected for data processing. Network consistency was evaluated by comparing the DIC between consistency and inconsistency models; a difference of less than five was considered evidence of adequate consistency, thus justifying the use of the consistency model for the final pooled estimates. Network graphs and comparison-adjusted funnel plots were generated using STATA.

## 3 Results

### 3.1 Literature screening process and results

**Figure 1:**
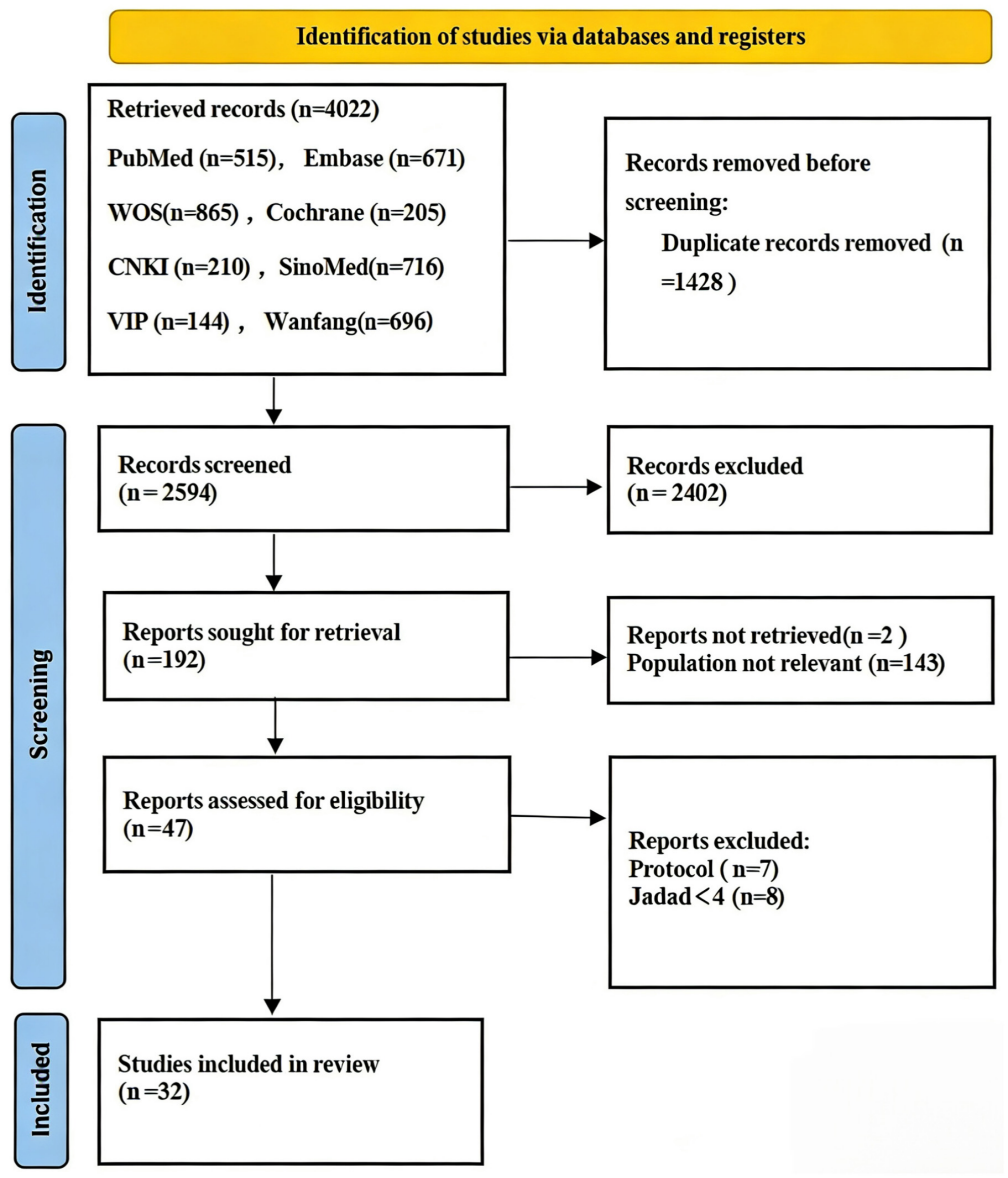
Flow diagram for the selection of the included studies.

A total of 4,022 records were retrieved from PubMed, Cochrane Library, Web of Science, EMBASE, SinoMed, CNKI, VIP, and Wanfang databases. After removing 1,428 duplicates, 2,594 records were screened. Based on titles and abstracts, 2,402 records were excluded, and 192 full-text articles were assessed for eligibility. Among them, 2 articles were unavailable, and 143 studies were excluded due to ineligible participants in the acute phase of disorders of consciousness. After exclusion of 7 protocols and 8 studies with a Jadad score < 4, 32 studies (25–30, 34–59) were finally included. The literature selection process is presented in Figure 1.

### 3.2 Basic information of included studies

Table 1 summarizes the baseline characteristics of the 32 included studies, involving 1,770 participants in total. Eight studies were conducted in Belgium, two in Italy, and the others in China. Among these studies, six did not report exact baseline age, three did not report gender ratio, and all studies met the predefined inclusion criteria. Regarding disease duration, 14 studies enrolled patients with a disease course of 1 to 3 months, 9 studies had a disease course longer than 3 months, and the remaining 9 studies did not report specific disease duration. For intervention characteristics, 5 studies adopted single-session stimulation, all of which were tDCS, while the others applied multi-day repeated stimulation.

**Table 1:** Characteristics of the included studies.

References	Country	Diagnosis (U/M)	Duration	Sex (F/M)	Age (Year)		Sample (*N*)	Intervention		
					EG	CG		EG	CG	Intensity
Xu et al. (25)	China	MCS	28–365 days	10/10	NA		20	rTMS	Sham	10 Hz; 20 min 10 sessions
Cheng et al. (26)	China	pDOC (19/9)	28–60 days	6/22	47.0 ± 16.3	46.5 ± 11.11	28	tDCS	Sham	2 mA; 20 min 20 sessions;
Xiong et al. (34)	China	pDOC (34/41)	61.53 ± 57.85 days	28/47	57.17 ± 15.25	58.8 ± 13.79	75	MNS_rTMS MNS	rTMS	10 Hz; 15–20 mA; 24 sessions
Thibaut et al. (28)	Belgium	pDOC (30/32)	37 ± 24.5 weeks	19/44	42 ± 12	45.5 ± 12	62	tDCS	Sham	2 mA; 20 min 20 sessions
Shen et al. (35)	China	UWS	NA	38/61	NA		99	rTMS	usual	20 Hz; 20 min 20 sessions
Fan et al. (36)	China	pDOC	49.0 ± 24.6 days	15/25	47.15 ± 10.73	50.65 ± 16.18	40	rTMS	Sham	20 Hz; 20 sessions
Bao et al. (30)	China	UWS	30.45 ± 13.8 days	14/84	44 ± 12	45 ± 15	100	acupuncture	usual	5 days/week, 30d ays;30min
Martens et al. (37)	Belgium	pDOC (17/29)	12 (5–47) months	19/27	46.33 ± 15.02		46	tDCS	Sham	1 mA; 20 min one session
Carrière et al. (38)	Belgium	MCS	NA	3/8	46 ± 14		13	tDCS	Sham	2 mA; 20 min one session
Martens et al. (39)	Belgium	pDOC (4/6)	7 ± 13 months	2/8	49 ± 20		10	tDCS	Sham	2 mA; 20 min one session
He et al. (40)	China	pDOC (3/3)	NA	2/4	NA		6	rTMS	Sham	20 Hz; 5 sessions
Thibaut et al. (41)	Belgium	MCS	NA	7/9	NA		16	tDCS	Sham	2 mA, 20 min 5 sessions
Cincotta et al. (42)	Italy	UWS	35 (9–85) months	4/7	NA		11	rTMS	Sham	20 Hz; 5 sessions
Thibaut et al. (43)	Belgium	pDOC (25/30)	43 ± 63 months	16/39	45.09 ± 18.39		30	tDCS	Sham	2 mA; 20 min one session
Barra et al. (27)	Belgium	pDOC (MCS)	8.8 ± 10.5 months	8/4	50.3 ± 14		5	tDCS	Sham	2 mA; 20 min one session
Estraneo et al. (44)	Italy	pDOC (7/6)	≥3 months	6/7	54.54 ± 21.64		13	tDCS	Sham	2 mA; 20 min 5 sessions
Martens et al. (45)	Belgium	MCS	NA	8/19	42 ± 14.47		27	tDCS	Sham	2 mA; 20 min 5 sessions
Wu et al. (46)	China	pDOC (8/7)	NA	6/9	47.63 ± 17.25		16	tDCS	Sham	2 mA; 20 min 10 sessions
Zhang et al. (47)	China	pDOC (11/15)	5.6 ± 4.6 months	NA	52.69 ± 20.17		26	tDCS	Sham	2 mA; 20 min 20 sessions
Zhang et al. (49)	China	UWS	≥3 months	14/34	56.13 ± 14.16	53.8 ± 12.38	48	rTMS	Sham	5 Hz; 20 min 40 sessions
Chen et al. (21)	China	pDOC	1–3 months	16/34	50.520 ± 13.86	52.60 ± 14.40	50	rTMS	Sham	10 Hz; 30 sessions
Xu et al. (50)	China	pDOC	3.8 ± 3.0 months	NA	NA		20	rTMS	Sham	10 Hz; 10 sessions
Zhou et al. (51)	China	pDOC (27/65)	42.76 ± 9.70 days	43/49	61.26 ± 6.08	60.09 ± 5.39	92	rTMS-MNS	MNS	3 Hz; 20 min 40 sessions
Ma et al. (53)	China	UWS	44.19 ± 3.61 days	26//44	33.5 ± 8.4	35.0 ± 7.8	70	rTMS	usual	0.5 Hz; 20 sessions
Zhu et al. (29)	China	pDOC	NA	17/66	42.3 ± 14.46	39.7 ± 13.12	83	HBO	usual	0.2 MPa; 20 min 20 sessions
Li et al. (30)	China	UWS	44.19 ± 3.61 days	37/43	30.42 ± 5.04	30.59 ± 4.52	80	rTMS	Sham	3 Hz; 20 min 20 sessions
Feng et al. (54)	China	MCS	28.53 ± 3.68 days	14/36	50.75 ± 4.51	51.23 ± 4.77	50	tDCS	usual	1–2 mA; 20 min 20 sessions
Feng et al. (55)	China	pDOC	NA	34/54	61.75 ± 5.71	61.52 ± 5.77	88	rTMS	usual	20 Hz; 7 min 72 sessions
Bao et al. (56)	China	UWS	27.45 ± 14.52 days	15/85	43 ± 16	45 ± 15	100	acupuncture	usual	1.5 mA; 30 min 30 sessions
Shen et al. (57)	China	UWS	48.05 ± 2.14 days	25/31	33.2 ± 10.15	34.7 ± 11.84	50	rTMS	Sham	3 Hz; 20 min 20 sessions
Lv et al. (58)	China	UWS	65.91 ± 17.48 days	20/42	NA		62	rTMS	usual	0.5 Hz; 4 weeks 20 sessions
Wang et al. (59)	China	MCS	35.68 ± 10.95 days	43/47	52.17 ± 10.94	50.7 ± 11.2	60	tDCS	Sham	1–2 mA; 20 min 96 sessions

### 3.3 Quality assessment of included studies

**Figure 2:**
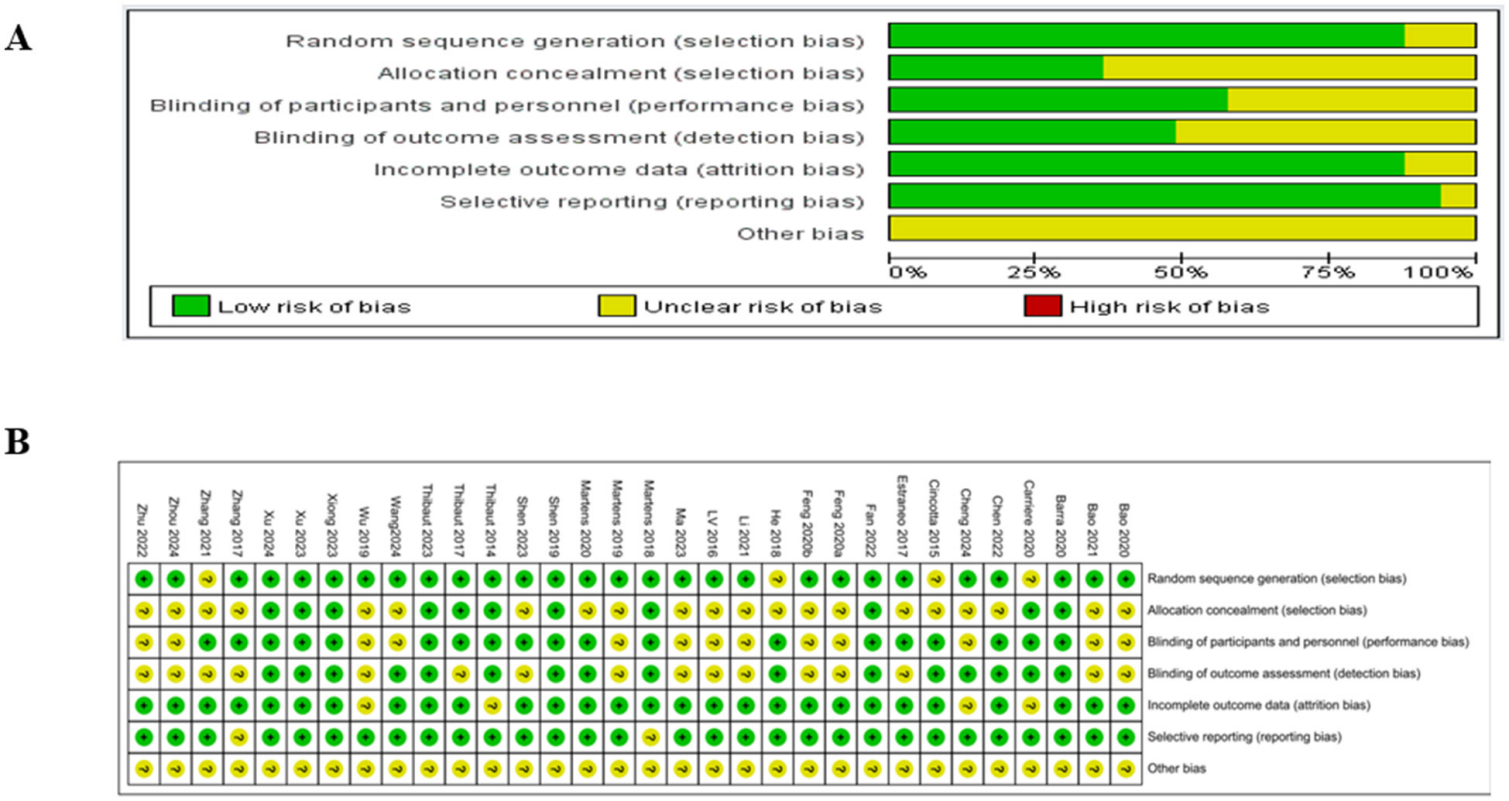
Methodological quality summary and quality graph. **(A)** Risk of bias graph. **(B)** Risk of bias summary.

Risk of bias assessment of the included studies showed that regarding random sequence generation, 29 studies reported explicit randomization methods (such as random number table, computer-generated sequence) and were rated as low risk. Only 3 studies (30, 49, 55) merely mentioned “random grouping” without specifying the method and were rated as unclear risk. With respect to allocation concealment, 11 studies adopted adequate measures (including sealed opaque envelopes) and were rated as low risk; the remaining 21 studies provided no details on allocation concealment and were rated as unclear risk. For blinding of participants and personnel, 18 studies achieved blinding and were rated as low risk, while 14 studies either did not report blinding details or could not perform blinding due to the intervention type. As for blinding of outcome assessment, 17 studies applied blinding to outcome assessors and were rated as low risk; the other 15 studies did not report relevant information and were rated as unclear risk. Concerning incomplete outcome data, 29 studies presented complete follow-up data and were rated as low risk. Only 4 studies (30, 38, 43, 46) had missing data or unclear descriptions of dropout and were rated as unclear risk. With regard to selective reporting, none of the studies showed selective outcome reporting, and all were rated as low risk. In terms of other bias, all 32 studies did not report potential sources of other bias and were rated as unclear risk. The detailed assessments are depicted in Figure 2.

### 3.4 Network meta-analysis

#### 3.4.1 The CRS-R total score

**Figure 3:**
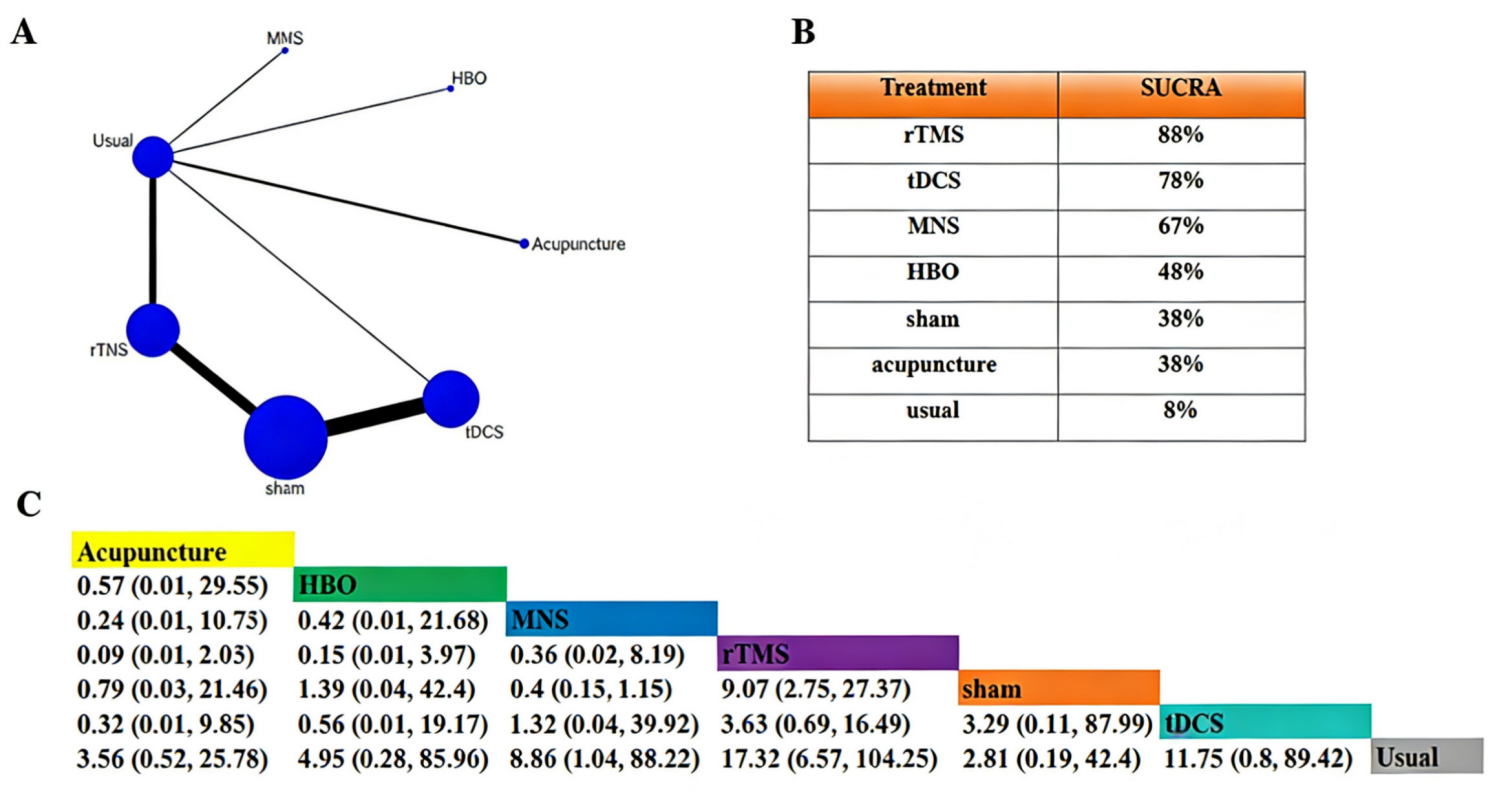
Network meta-analysis of the CRS-R total score. **(A)** Network plot (overall). **(B)** SUCRA ranking plot (overall). **(C)** League table (overall).

The included studies covered a total of 7 intervention measures: rTMS (*n* = 15), tDCS (*n* = 12), MNS (*n* = 2), HBO (*n* = 1), acupuncture (*n* = 2), usual treatment (*n* = 21), and sham treatment (*n* = 9). Among these interventions, rTMS, usual treatment, tDCS, and sham stimulation had relatively larger sample sizes. The most frequent comparisons were between rTMS and usual treatment, rTMS and sham treatment, and tDCS and sham stimulation. The network diagram is shown in Figure 3A. According to the SUCRA value, the results indicated that rTMS (SUCRA = 88%) ranked the highest in improving the CRS-R score, followed by tDCS (SUCRA = 78%) and MNS combination (SUCRA = 67%). Specific values are shown in Figure 3B. Compared with usual treatment, rTMS [MD = 17.32, 95% CrI (6.57, 104.25)], tDCS [MD = 11.75, 95% CrI (0.8, 89.42)], HBO [MD = 4.95, 95% CrI (0.28, 85.96)], MNS [MD = 8.86, 95% CrI (1.04, 88.22)], acupuncture [MD = 3.56, 95% CrI (0.61, 40.45)], and sham stimulation [MD = 7.77, 95% CrI (0.52, 25.78)] could significantly improve the total score of CRS-R (Figure 3C). Compared with sham stimulation, rTMS [MD = 6.67, 95% CrI (2.05, 20.78)] and tDCS [MD = 0.18, 95% CrI (0.06, 0.53)] could significantly improve the total score of CRS-R.

### 3.5 The Subgroup analyses

#### 3.5.1 Subgroup analysis for MCS

**Figure 4:**
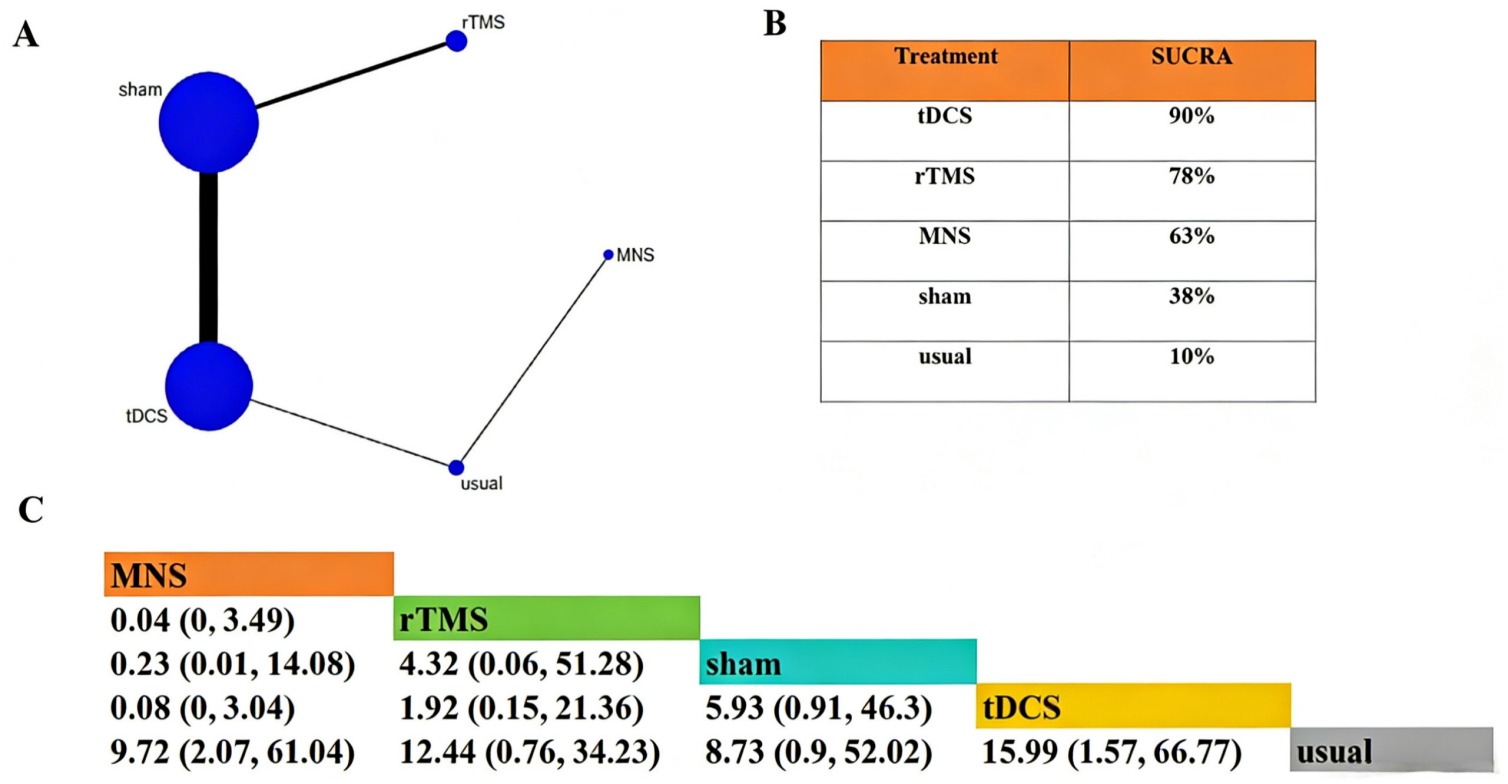
Network meta-analysis of the CRS-R total score for MCS. **(A)** Network plot (MCS). **(B)** SUCRA ranking plot (MCS). **(C)** League table (MCS).

The included studies covered a total of 5 intervention measures: rTMS, tDCS, MNS, usual treatment, and sham treatment. The sample sizes of tDCS and sham stimulation were larger. The network diagram is shown in Figure 4A. According to the SUCRA value, the results indicated that tDCS (SUCRA = 90%) ranked the highest in improving the CRS-R score, followed by rTMS (SUCRA = 78%) and MNS (SUCRA = 63%). Specific values are shown in Figure 4B. Compared with usual treatment, tDCS [MD = 15.99, 95% CrI (1.57, 66.77)], rTMS (MD = 12.44, 95% CrI (0.76, 34.23), MNS [MD = 9.72, 95% CrI (2.07, 61.04)], and sham stimulation [MD = 8.73, 95% CrI(0.9, 52.02)] could significantly improve the total score of CRS-R. Compared with sham stimulation, rTMS [MD = 4.32, 95% CrI (0.06, 51.28)] and tDCS [MD = 5.93, 95% CrI (0.91, 46.3)] could significantly improve the total score of CRS-R (Figure 4C).

#### 3.5.2 Subgroup analysis for UWS

**Figure 5:**
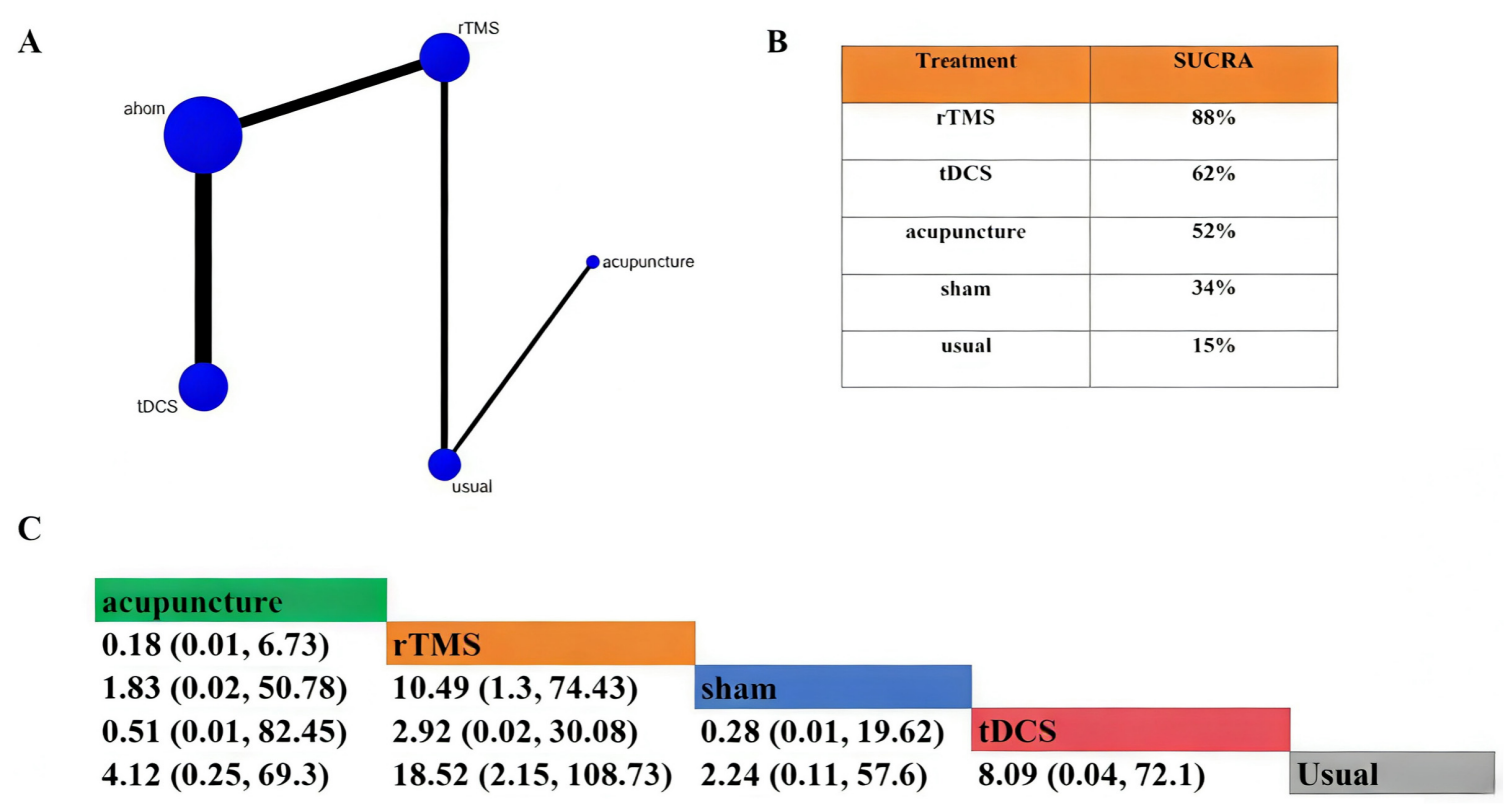
Network meta-analysis of the CRS-R total score for UWS. **(A)** Network plot (UWS). **(B)** SUCRA ranking plot (UWS). **(C)** League table (UWS).

The included studies covered a total of 5 intervention measures: rTMS, tDCS, acupuncture, usual treatment, and sham treatment. The sample sizes of rTMS, usual treatment, and tDCS, as well as sham stimulation were larger. The network diagram is shown in Figure 5A. According to the SUCRA value, the results indicated that rTMS (SUCRA = 88%) ranked the highest in improving the CRS-R score, followed by tDCS (SUCRA = 62%) and acupuncture (SUCRA = 52%). Specific values are shown in Figure 5B. Compared with usual treatment, rTMS (MD = 18.52 95% CrI (2.15, 108.73), acupuncture [MD = 4.12, 95% CrI (0.25, 69.3)], tDCS [MD = 8.09, 95% CrI (0.04, 72.1)], and sham stimulation [MD = 2.24, 95% CrI(0.11, 57.6)] could significantly improve the total score of CRS-R (Figure 5C). Compared with sham stimulation, rTMS significantly improved the total CRS-R score [MD = 10.49, 95% CrI (1.3, 74.43)]. For tDCS, a statistically significant but minimal effect was observed [MD = 0.28, 95% CrI (0.01, 19.62)].

### 3.5 Publication bias

**Figure 6:**
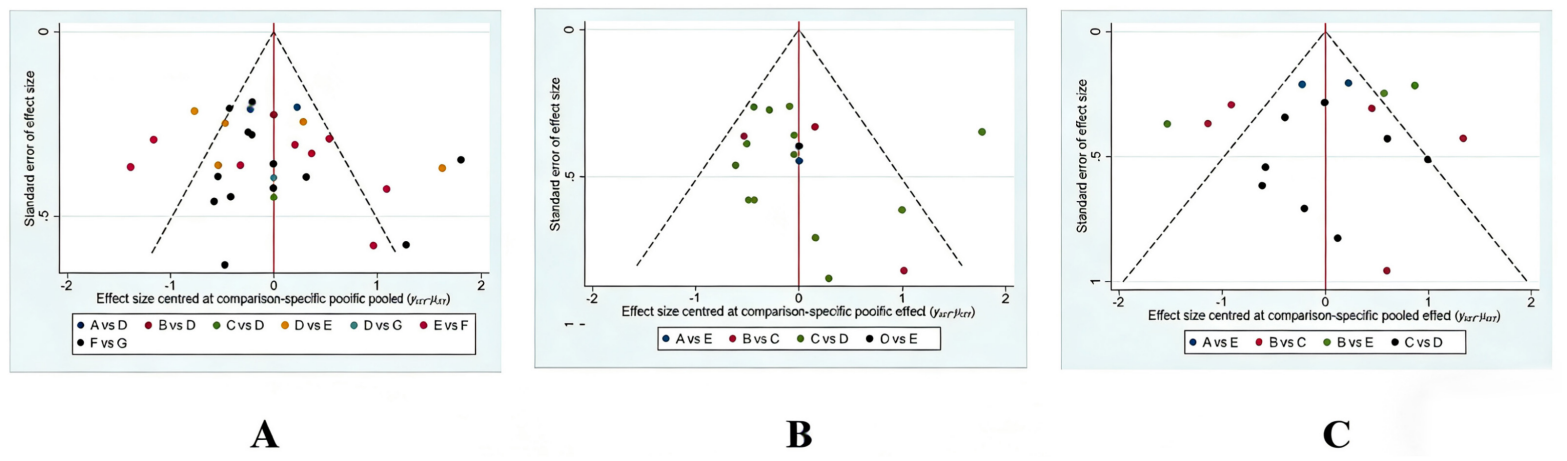
The comparison-adjusted funnel plots. **(A)** Overall analysis. **(B)** MCS subgroup. **(C)** UWS subgroup.

The results of comparison-adjusted funnel plots (Figure 6) were corrected for publication bias by comparison. All the included randomized controlled trials had a certain degree of asymmetry and heterogeneity, suggesting that the included studies may have a certain publication bias or small sample effect.

## 4 Discussion

Patients with pDOC represent a major clinical challenge in neurocritical care and rehabilitation medicine (60). Previous studies have highlighted the potential of neuromodulation as an important non-pharmacological approach for pDOC, including non-invasive modalities such as tDCS, rTMS, and MNS, as well as invasive strategies including deep brain stimulation and spinal cord stimulation (61). However, few network meta-analyses have directly compared the relative effectiveness of different non-invasive interventions for pDOC. This analysis included five non-invasive approaches—rTMS, tDCS, MNS, HBO, and acupuncture—to systematically evaluate their effects on CRS-R scores in patients with pDOC, aiming to inform evidence-based clinical decision-making.

This study demonstrated that all five interventions significantly increased CRS-R scores in pDOC patients compared with the usual control group, suggesting that these treatments may be associated with improved consciousness. Based on SUCRA probability rankings, rTMS ranked most favorably for improving CRS-R total scores, followed by tDCS, MNS, HBO, and acupuncture. Subgroup analyses suggested that tDCS was ranked first in the MCS subgroup, whereas rTMS was top in the UWS subgroup. Within the limitations of the available evidence, rTMS and tDCS appeared to demonstrate relatively favorable effects on behavioral consciousness in pDOC and may provide preliminary support for clinical decision-making. Specifically, rTMS may represent a preferential option for UWS, whereas tDCS may be more suitable for MCS. However, given the limited number and small sample sizes of included studies in subgroups, these results should be interpreted with caution and are not sufficiently robust to serve as strong evidence for clinical practice. The observed subgroup differences may be related to distinct pathophysiological profiles between UWS and MCS. UWS is typically characterized by widespread thalamocortical disconnection and severely diminished cortical functional connectivity, impairing integration between arousal and consciousness-related networks (62). Such extensive network disruption may reduce neural responsiveness to external stimulation. Theoretically, high-frequency rTMS may modulate cortical excitability through electromagnetic effects and indirectly engage thalamocortical circuits, thereby potentially enhancing activity within consciousness-relevant networks (63, 64). By contrast, MCS patients retain partial but inefficient cortical connectivity (65); tDCS may exert cortical modulation by altering neuronal membrane potentials and regulating residual functional networks (66, 67). However, the present study did not include neuroimaging or neurophysiological assessments; thus, all mechanistic inferences remain theoretical and lack direct empirical confirmation. Furthermore, 5 of 13 tDCS studies adopted single-session protocols, introducing substantial heterogeneity that may compromise the stability of efficacy evaluation. The relatively low rankings of acupuncture and HBO may reflect their indirect mechanisms of action, considerable interindividual variability, and limited sample sizes. Further well-designed clinical investigations are required to validate these observations. Collectively, large-scale, multicenter randomized controlled trials with standardized treatment protocols and prolonged follow-up are urgently needed to verify the reliability of these findings.

In the clinical management of pDOC, safety and efficacy are equally important. According to clinical guidelines, uncontrolled active epilepsy is a common contraindication for both rTMS and tDCS. Intracranial metallic implants represent an absolute contraindication for rTMS, whereas such conditions require rigorous precaution and individualized risk assessment for tDCS. Patients with skull defects should undergo thorough evaluation before receiving transcranial neuromodulation. For patients with contraindications or poor tolerance to rTMS and tDCS, MNS, HBO, and acupuncture are reasonable alternatives. As a peripheral neuromodulatory technique, MNS avoids intracranial risks, while HBO and acupuncture are associated with low adverse event rates and favorable safety profiles. Combining efficacy and safety considerations, this study proposes a stratified clinical strategy for pDOC: patients without contraindications may receive subtype-specific interventions, while those with contraindications or intolerance may benefit from MNS, HBO, or acupuncture as part of comprehensive rehabilitation.

Importantly, this study relied exclusively on the CRS-R total score as the primary outcome, which reflects consciousness level solely based on behavioral function rather than directly assessing neural activity or subjective awareness. Although CRS-R is an internationally recognized tool for diagnosis and outcome assessment in pDOC (68, 69), comprehensive evaluation of consciousness recovery requires a multimodal, multidimensional assessment system (70). Clinically meaningful recovery is reflected not only by increased CRS-R scores but also by transitions in consciousness states, such as emergence from UWS to MCS or from MCS to full wakefulness, alongside improvements in functional independence and quality of life. Future studies should integrate standardized CRS-R assessment, neuroimaging modalities including functional magnetic resonance imaging, neurophysiological markers such as electroencephalography, and functional and quality-of-life scales to achieve objective and comprehensive evaluation of consciousness recovery.

### Study limitations

Several limitations of this study should be acknowledged. First, the outcome was limited to CRS-R scores without multimodal objective markers, and CRS-R may be influenced by short-term fluctuations and spontaneous neurological recovery, potentially introducing bias. Second, significant heterogeneity existed across included studies in terms of stimulation parameters, intervention duration, frequency, and treatment courses, limiting the comparability of efficacy and constraining mechanistic interpretations. Third, patients varied in disease duration and etiology, which were not adjusted for in meta-regression, potentially introducing confounding bias. Fourth, some studies were derived from Chinese-language databases, which may increase publication bias and limit generalizability. Fifth, the lack of long-term follow-up data precluded evaluation of the durability of intervention effects.

To address these limitations, future research should adopt standardized intervention protocols, extend follow-up duration, incorporate multidimensional outcome measures, and enroll more representative study populations. Unification of key technical parameters and outcome criteria, combined with objective neuroimaging and neurophysiological biomarkers, will enhance the quality of evidence, depth of mechanistic understanding, and generalizability of conclusions, thereby supporting individualized neurorehabilitation strategies for patients with pDOC.

## 5 Conclusion

In conclusion, rTMS, tDCS, MNS, HBO, and acupuncture may improve CRS-R scores and potentially enhance consciousness recovery in patients with pDOC. rTMS and tDCS may show relatively superior efficacy. In particular, tDCS may be more favorable for MCS patients, whereas rTMS may be more suitable for UWS patients.

## Data Availability

The original contributions presented in the study are included in the article/supplementary material, further inquiries can be directed to the corresponding author.

## References

[1] Ma H Fan S Xu Z Wan X Yang Q Yin Y . Trigeminal nerve stimulation for prolonged disorders of consciousness: A randomized double-blind sham-controlled study. Brain Stimul. (2023) 16:819–27. doi: 10.1016/j.brs.2023.05.002 37182683

[2] Song M Yang Y Yang Z Cui Y Yu S He J . Prognostic models for prolonged disorders of consciousness: an integrative review. Cell Mol Life Sci. (2020) 77:3945–61. doi: 10.1007/s00018-020-03512-z 32306061 PMC11104990

[3] Schnakers C Monti MM. Disorders of consciousness after severe brain injury: Therapeutic options. Curr Opin Neurol. (2017) 30:573–9. doi: 10.1097/WCO.0000000000000495 28901969

[4] Plosnić G RaguŽ M Deletis V Chudy D. Dysfunctional connectivity as a neurophysiologic mechanism of disorders of consciousness: a systematic review. Front Neurol. (2023) 17:1166187. 187 doi: 10.3389/fnins.2023.1166187 37539385 PMC10394244

[5] Jang SH Yeo SS Cho MJ Chung WK. Correlation between thalamocortical tract and default mode network with consciousness levels in hypoxic-ischemic brain injury patients: a comparative study using the coma recovery scale-revised. Med Sci Monit. (2024) 30:e943802. doi: 10.12659/MSM.943802 38741355 PMC11409902

[6] Giacino JT Katz DI Schiff ND Whyte J Ashman EJ Ashwal S . Practice guideline update recommendations summary: disorders of consciousness. Neurology. (2018) 91:450–60. doi: 10.1212/WNL.0000000000005926 30089618 PMC6139814

[7] Kondziella D Bender A Diserens K van Erp W Estraneo A Formisano R . EAN panel on coma, disorders of consciousness. Eur J Neurol. (2020) 27:741–56. doi: 10.1111/ene.14151 32090418

[8] Thibaut A Schiff N Giacino J Laureys S Gosseries O. Therapeutic interventions in patients with prolonged disorders of consciousness. Lancet Neurol. (2019) 18:600–14. doi: 10.1016/S1474-4422(19)30031-6 31003899

[9] Wan X Zhang Y Li Y Song W. An update on noninvasive neuromodulation in the treatment of patients with prolonged disorders of consciousness. CNS Neurosci Ther. (2024) 30:e14757. doi: 10.1111/cns.14757 38747078 PMC11094579

[10] Hintze TD Small CE Montgomery J Reveles KR Hafeez S Barthol CA. Comparison of amantadine, modafinil, and standard of care in the acute treatment of disorders of consciousness after severe traumatic brain injury. Clin Neuropharmacol. (2022) 45:1–6. doi: 10.1097/WNF.0000000000000487 35029862

[11] Girard Pepin R Seyfzadeh F Williamson D Gosseries O Duclos C. Pharmacological therapies for early and long-term recovery in disorders of consciousness: current knowledge and promising avenues. Expert Rev Neurother. (2025) 25:613–33. doi: 10.1080/14737175.2025.2500757 40336212

[12] Gatto LAM Demartini Z Jr Telles JPM Figueiredo EG. Does amantadine improve cognitive recovery in severe disorders of consciousness after aneurysmal subarachnoid hemorrhage? A double-blind placebo-controlled study. Clin Neurol Neurosurg. (2024) 237:108135. doi: 10.1016/j.clineuro.2024.108135 38330801

[13] Machado C Estévez M Rodríguez R Pérez-Nellar J Chinchilla M DeFina P . Zolpidem arousing effect in persistent vegetative state patients: autonomic, EEG and behavioral assessment. Curr Pharm Des. (2014) 20:4185–202. 24025063

[14] Thonnard M Gosseries O Demertzi A Lugo Z Vanhaudenhuyse A Bruno MA . Effect of zolpidem in chronic disorders of consciousness: a prospective open-label study. Funct Neurol. (2013) 28:259–64. 24598393 PMC3951253

[15] Davis CK Arruri V Joshi P Vemuganti R. Non-pharmacological interventions for traumatic brain injury. J Cereb Blood Flow Metab. (2024) 44:641–59. doi: 10.1177/0271678X241234770 38388365 PMC11197135

[16] Gao Y Bai J Gu Z Xie Y An W Liu Z . Noninvasive neuromodulation for disorders of consciousness: an updated systematic review and meta-analysis. Crit Care. (2025) 29:269. doi: 10.1186/s13054-025-05429-0 40611258 PMC12224763

[17] Chinese Society of Rehabilitation Medicine Professional Committee on Disorders of Consciousness Rehabilitation. Chinese expert consensus on comprehensive rehabilitation and non-invasive neuromodulation for arousal in patients with chronic disorders of consciousness. Chin J Rehabil Theor Pract (2025) 31:1365–75. doi: 10.3969/j.issn.1006-9771.2025.12.001

[18] Fan W Fan Y Liao Z Yin Y. Effect of transcranial direct current stimulation on patients with disorders of consciousness: a systematic review and meta-analysis. Am J Phys Med Rehabil. (2023) 102:1102–10. doi: 10.1097/PHM.0000000000002290 37205736

[19] Yang Z Yue T Zschorlich VR Li D Wang D Qi F. Behavioral effects of repetitive transcranial magnetic stimulation in disorders of consciousness: a systematic review and meta-analysis. Brain Sci. (2023) 13:1362. doi: 10.3390/brainsci13101362 37891731 PMC10605911

[20] Gu C Shang H Kong Y Peng M Jiang H Wang S . Safety of median nerve electrical stimulation in disorders of consciousness: A systematic review and meta-analysis of randomized controlled trials. PLoS ONE. (2025) 20:e0324046. doi: 10.1371/journal.pone.0324046 40743130 PMC12312889

[21] Huang Z Chen Y Xiao Q Kuang W Liu K Jiang Y . Effect of acupuncture for disorders of consciousness in patients with stroke: a systematic review and meta-analysis. Front Neurol. (2022) 13:930546. doi: 10.3389/fneur.2022.930546 36277925 PMC9581330

[22] Li G Wang B Fan S Liu S Shao L Li C . The effect of acupuncture combined with hyperbaric oxygenation compared with hyperbaric oxygenation alone for patients with traumatic brain injury: a systematic review and meta-analysis. Front Neurol. (2025) 16:1538740. doi: 10.3389/fneur.2025.1538740 40386021 PMC12083083

[23] Liu Z Zhang X Yu B Wang J Lu X. Effectiveness on level of consciousness of non-invasive neuromodulation therapy in patients with disorders of consciousness: a systematic review and meta-analysis. Front Hum Neurosci. (2023) 17:1129254. doi: 10.3389/fnhum.2023.1129254 37292582 PMC10246452

[24] Caselli S Leonardi M Magnani FG Cacciatore M Barbadoro F Ippoliti C . Comparing the different sets of item-level diagnostic criteria of the coma recovery scale-revised (CRS-R): a measurement-based approach driven by RASCH analysis. Arch Phys Med Rehabil (2025) 106:1028–38.e8. doi: 10.1016/j.apmr.2024.12.009 39706237

[25] Xu C Yuan Z Chen Z Liao Z Li S Feng Y . Perturbational complexity index in assessing responsiveness to rTMS treatment in patients with disorders of consciousness: a cross-over randomized controlled trial study. J Neuroeng Rehabil. (2024) 21:167. doi: 10.1186/s12984-024-01455-1 39300529 PMC11411826

[26] Cheng XR Zhang YB Sun DJ Peng XY Bao YC Zhang F . Long-term repetitive transcranial direct current stimulation in patients with disorders of consciousness: a preliminary study. Brain Inj. (2024) 38:68–75. doi: 10.1080/02699052.2024.2304872 38329075

[27] Barra A Rosenfelder M Mortaheb S Carriere M Martens G Bodien YG . Transcranial pulsed-current stimulation versus transcranial direct current stimulation in patients with disorders of consciousness: a pilot, sham-controlled cross-over double-blind study. Brain Sci. (2022) 12:1–10. doi: 10.3390/brainsci12040429 PMC9031379 35447961

[28] Thibaut A Fregni F Estraneo A Fiorenza S Noe E Llorens R . Sham-controlled randomized multicentre trial of transcranial direct current stimulation for prolonged disorders of consciousness. Eur J Neurol. (2023) 30:3016–31. doi: 10.1111/ene.15974 37515394

[29] Zhu X Hu C Huang D Jiang W. Observation on the awakening effect of hyperbaric oxygen therapy in patients with disorders of consciousness due to severe craniocerebral injury. Chin J Geriatr Care (2022) 20:39–40, 44.

[30] Bao YC Zhang F Li Q Liu M Cheng XR Zhang YB . Xingnao Kaiqiao acupuncture on promoting wake-up of vegetative state after brain injury. Zhongguo Zhen Jiu. (2021) 41:1225–8. doi: 10.13703/j.0255-2930.20201101-k0002 34762375

[31] Rethlefsen ML Kirtley S Waffenschmidt S Ayala AP Moher D Page MJ . PRISMA-S: an extension to the PRISMA statement for reporting literature searches in systematic reviews. Syst Rev. (2021) 10:1–12. doi: 10.1186/s13643-020-01542-z 33499930 PMC7839230

[32] Jadad AR Moore RA Carroll D Jenkinson C Reynolds DJM Gavaghan DJ . Assessing the quality of reports of randomized clinical trials: is blinding necessary? Control Clin Trials. (1996) 17:1–12. doi: 10.1016/0197-2456(95)00134-4 8721797

[33] Higgins JPT Altman DG Gøtzsche PC Jüni P Moher D Oxman AD . The Cochrane collaboration's tool for assessing risk of bias in randomised trials. BMJ. (2011) 343:d5928. doi: 10.1136/bmj.d5928 22008217 PMC3196245

[34] Xiong Q Le K Tang Y Ye W Wang Y Zhong Y . Effect of single and combined median nerve stimulation and repetitive transcranial magnetic stimulation in patients with prolonged disorders of consciousness: a prospective, randomized, single-blinded, controlled trial. Front Aging Neurosci. (2023) 15:1–10. doi: 10.3389/fnagi.2023.1112768 PMC10164991 37168716

[35] Shen L Huang Y Liao Y Yin X Huang Y Ou J . Effect of high-frequency repetitive transcranial magnetic stimulation over M1 for consciousness recovery after traumatic brain injury. Brain Behav. (2023) 13:e29281. doi: 10.1002/brb3.2971 PMC10176007 36977194

[36] Fan J Zhong Y Wang H Aierken N He R. Repetitive transcranial magnetic stimulation improves consciousness in some patients with disorders of consciousness. Clin Rehabil. (2022) 36:916–25. doi: 10.1177/02692155221089455 35322709

[37] Martens G Kroupi E Bodien Y Frasso G Annen J Cassol H . Behavioral and electrophysiological effects of network-based frontoparietal tDCS in patients with severe brain injury: a randomized controlled trial. Neuroimage Clin. (2020) 28:1–12. doi: 10.1016/j.nicl.2020.102426 PMC7511767 32977212

[38] Carriere M Mortaheb S Raimondo F Annen J Barra A Binda Fossati MC . Neurophysiological correlates of a single session of prefrontal tdcs in patients with prolonged disorders of consciousness: a pilot double-blind randomized controlled study. Brain Sci. (2020) 10:1–17. doi: 10.3390/brainsci10070469 PMC7408434 32708119

[39] Martens G Fregni F Carrière M Barra A Laureys S Thibaut A. Single tDCS session of motor cortex in patients with disorders of consciousness: a pilot study. Brain Inj. (2019) 33:1679–83. doi: 10.1080/02699052.2019.1667537 31523995

[40] He F Wu M Meng F Hu Y Gao J Chen Z . Effects of 20 Hz repetitive transcranial magnetic stimulation on disorders of consciousness: a resting-state electroencephalography study. Neural Plast. (2018) 2018:5036184. doi: 10.1155/2018/5036184 29770146 PMC5889874

[41] Thibaut A Wannez S Donneau AF Chatelle C Gosseries O Bruno MA . Controlled clinical trial of repeated prefrontal tDCS in patients with chronic minimally conscious state. Brain Inj. (2017) 31:466–74. doi: 10.1080/02699052.2016.1274776 28281845

[42] Cincotta M Giovannelli F Chiaramonti R Bianco G Godone M Battista D . No effects of 20 Hz-rTMS of the primary motor cortex in vegetative state: a randomised, sham-controlled study. Cortex. (2015) 71:368–76. doi: 10.1016/j.cortex.2015.07.027 26301875

[43] Thibaut A Bruno MA Ledoux D Demertzi A Laureys S. tDCS in patients with disorders of consciousness: sham-controlled randomized double-blind study. Neurology. (2014) 82:1112–8. doi: 10.1212/WNL.0000000000000260 24574549

[44] Estraneo A Pascarella A Moretta P Masotta O Fiorenza S Chirico G . Repeated transcranial direct current stimulation in prolonged disorders of consciousness: a double-blind cross-over study. J Neurol Sci. (2017) 375:464–70. doi: 10.1016/j.jns.2017.02.036 28320187

[45] Martens G Lejeune N O'Brien AT Fregni F Martial C Wannez S . Randomized controlled trial of home-based 4-week tDCS in chronic minimally conscious state. Brain Stimul. (2018) 11:982–90. doi: 10.1016/j.brs.2018.04.021 29759943

[46] Wu M Yu Y Luo L Wu Y Gao J Ye X . Efficiency of repetitive transcranial direct current stimulation of the dorsolateral prefrontal cortex in disorders of consciousness: a randomized sham-controlled study. Neural Plast. (2019) 2019:7089543. doi: 10.1155/2019/7089543 31308848 PMC6594311

[47] Zhang Y Song W Du J Huo S Shan G Li R. Transcranial direct current stimulation in patients with prolonged disorders of consciousness: combined behavioral and event-related potential evidence. Front Neurol. (2017) 8:620. doi: 10.3389/fneur.2017.00620 29209270 PMC5702306

[48] Guo ZQ Jiang H Huang Y Gu HM Wang WB Chen TD. Early complementary acupuncture improves the clinical prognosis of traumatic brain edema: a randomized controlled trial. Medicine. (2022) 101:e28959. doi: 10.1097/MD.0000000000028959 35212308 PMC8878911

[49] Zhang XH Han P Zeng YY Wang YL Lv HL. The clinical effect of repetitive transcranial magnetic stimulation on the disturbance of consciousness in patients in a vegetative state. Front Neurosci. (2021) 15:1–8. doi: 10.3389/fnins.2021.647517 33994925 PMC8119637

[50] Xu C Wu W Zheng X Liang Q Huang X Zhong H . Repetitive transcranial magnetic stimulation over the posterior parietal cortex improves functional recovery in nonresponsive patients: a crossover, randomized, double-blind, sham-controlled study. Front Neurol. (2023) 14:1–9. doi: 10.3389/fneur.2023.1059789 PMC9978157 36873436

[51] Zhou Q Sun J. Analysis on application effect of hrTMS combined with MSS in patients with prolonged disorders of consciousness after severe traumatic brain injury. Chongqing Med J. (2024) 53:93–7.

[52] Ma H Zhang R Xiong J Zhang P. The awakening effect of low-frequency repetitive transcranial magnetic stimulation on patients in persistent vegetative state after craniocerebral injury. J Chin Physician (2023) 25:1–6.

[53] Li Y Li J Wang D Zhen Z. Effects of high-frequency repetitive transcranial magnetic stimulation on neuroelectrophysiology and cerebrospinal fluid excitatory amino acid levels in patients with disorders of consciousness after severe craniocerebral injury. J Clin Med Pract. (2021) 25:57–60.

[54] Feng F Sun X Ju F Chen C Hui N Du X . Clinical effect of transcranial direct current stimulation in the treatment of consciousness disorder after severe craniocerebral injury and its influence on patients' neurological function. Clin Res Pract. (2020) 5:7–8, 16.

[55] Feng F. The role of repetitive transcranial magnetic stimulation combined with usual wake-up therapy in the treatment of patients with disturbance of consciousness after craniocerebral injury. Shaanxi Med J. (2020) 49:442–5.

[56] Bao Y Zhang F Li Q Liu M Cheng X Zhang Y . Midnight-noon ebb-flow acupuncture combined with rehabilitation therapy for severe craniocerebral trauma patients with vegetative state: a randomized controlled trial. Chin Acupunct Moxibust. (2020) 40:234–8. 32270632 10.13703/j.0255-2930.20191028-k0001

[57] Shen L Ouyang H Yang C Lin Z Mou Z Wang H . Therapeutic effect of high-frequency repetitive transcranial magnetic stimulation on arousal in patients with disorders of consciousness after severe craniocerebral injury. Chin J Rehabil Med. (2019) 34:1411–7.

[58] Lv C Fei Z Hu X Luo P Zhang L Li S . Awakening efficacy of low frequency repetitive transcranial magnetic stimulation for the patients with vegetative state after craniocerebral injury. China Med Herald (2016) 13:69–72.

[59] Wang S Lu W Li Q Sun L Liu M. Effect of transcranial direct current stimulation with different stimulation parameters on patients with minimal disturbance of consciousness after craniocerebral injury. Med J Wuhan Univ. (2024) 1–7.

[60] Ragazzoni A Cincotta M Giovannelli F Cruse D Young GB Miniussi C . Clinical neurophysiology of prolonged disorders of consciousness: From diagnostic stimulation to therapeutic neuromodulation. Clin Neurophysiol. (2017) 128:1629–46. doi: 10.1016/j.clinph.2017.06.037 28728060

[61] Vitello MM Laureys S Thibaut A Gosseries O. Non-pharmacologic interventions in disorders of consciousness. Handb Clin Neurol. (2025) 207:197–216. doi: 10.1016/B978-0-443-13408-1.00007-5 39986722

[62] Boly M Garrido MI Gosseries O Bruno MA Boveroux P Schnakers C . Preserved feedforward but impaired top-down processes in the vegetative state. Science. (2011) 332:858–62. doi: 10.1126/science.1202043 21566197

[63] Edlow BL Claassen J Schiff ND Greer DM. Recovery from disorders of consciousness: mechanisms, prognosis and emerging therapies. Nat Rev Neurol. (2021) 17:135–56. doi: 10.1038/s41582-020-00428-x 33318675 PMC7734616

[64] Sheng R Chen C Chen H Yu P. Repetitive transcranial magnetic stimulation for stroke rehabilitation: insights into the molecular and cellular mechanisms of neuroinflammation. Front Immunol. (2023) 14:1197422. doi: 10.3389/fimmu.2023.1197422 37283739 PMC10239808

[65] Li H Zhang X Sun X Dong L Lu H Yue S . Functional networks in prolonged disorders of consciousness. Front Neurosci. (2023) 17:1113695. doi: 10.3389/fnins.2023.1113695 36875660 PMC9981972

[66] Nitsche MA Paulus W. Excitability changes induced in the human motor cortex by weak transcranial direct current stimulation. J Physiol. (2000) 527:633–9. doi: 10.1111/j.1469-7793.2000.t01-1-00633.x 10990547 PMC2270099

[67] Stagg CJ Nitsche MA. Physiological basis of transcranial direct current stimulation. Neuroscientist. (2011) 17:37–53. doi: 10.1177/1073858410386614 21343407

[68] Giacino JT Kalmar K Whyte J. The JFK coma recovery scale-revised: measurement characteristics and diagnostic utility. Arch Phys Med Rehabil. (2004) 85:2020–9. doi: 10.1016/j.apmr.2004.02.033 15605342

[69] Schnakers C Vanhaudenhuyse A Giacino JT Ventura M Boly M Majerus S . Diagnostic accuracy of the vegetative and minimally conscious state: clinical consensus versus standardized neurobehavioral assessment. BMC Neurol. (2009) 9:35. doi: 10.1186/1471-2377-9-35 19622138 PMC2718857

[70] Zhang B Darji N Giacino JT. Definitions, diagnostic criteria, and clinical assessment scales in disorders of consciousness. Handb Clin Neurol. (2025) 207:1–13. doi: 10.1016/B978-0-443-13408-1.00011-7 39986716

